# The Association Between Serum Estradiol Levels on hCG Trigger Day and Live Birth Rates in Non-PCOS Patients: A Retrospective Cohort Study

**DOI:** 10.3389/fendo.2022.839773

**Published:** 2022-05-03

**Authors:** Xiaoyuan Xu, Aimin Yang, Yan Han, Wei Wang, Guimin Hao, Na Cui

**Affiliations:** Hebei Key Laboratory of Infertility and Genetics, Hebei Clinical Research Center for Birth Defects, Department of Reproductive Medicine, Second Hospital of Hebei Medical University, Shijiazhuang, China

**Keywords:** *in vitro* fertilization, live birth rate, estradiol, assisted reproductive technology, fresh embryo transfer

## Abstract

**Objective:**

To retrospectively analyze the association of serum estradiol (E2) levels on human chorionic gonadotropin (hCG) trigger day and live birth rates (LBRs) in women undergoing fresh embryo transfer and not exhibiting polycystic ovary syndrome.

**Design:**

Retrospective cohort study.

**Methods:**

Analysis of 13,950 patients who had fresh embryo transfer between December 2013 and December 2019. The main outcome measurement was LBRs. Multivariable regression analysis was performed to investigate associations between E2 levels on the hCG trigger day and LBRs. Stratification analysis was performed to test for effect modification in subgroups. Furthermore, a two-piecewise linear regression model was established to find nonlinear relationships.

**Results:**

Multivariable regression analysis showed a significant association between serum E2 levels on the hCG trigger day and LBRs, adjusting for covariates [relative risk (RR) 1.027, 95% confidence interval (CI) 1.007, 1.049]. Stratification analysis showed that the LBRs were positively associated (RR 1.052, 95% CI 1.004, 1.102) with every 1 ng/ml increase of serum E2 on the hCG trigger day for the subgroup with low antral follicle counts on the trigger day. Specifically, a two-piecewise linear regression model showed that there was a positive association (RR 1.188, 95% CI 1.057, 1.334) between serum E2 and LBR for every increase of 1 ng/ml E2 when the concentration of serum E2 was lower than 2.1 ng/ml. However, there was no significant association (RR 1.002, 95% CI 0.971, 1.032) between E2 levels and LBRs when the concentration of E2 was higher than the 2.1ng/ml inflection point.

**Conclusions:**

Serum E2 levels on the hCG trigger day were segmentally connected with LBRs.

## Introduction

Assisted reproductive technology (ART) has become an integral part of infertility treatment and the proportion of infants born after ART exeeds5% in some countries (1). Controlled ovulation hyperstimulation (COH) plays a crucial role in ART, inducing the maturation of many follicles and increasing the number of retrieved oocytes (2). Recently, attention has turned to hyper-physiological estrogen levels resulting from COH. High estradiol (E2) levels have been shown to affect fertilization because of the rupture of the zona pellucida (3, 4), and adverse effects on endometrial receptivity and embryo implantation (5, 6). High E2 levels are also related to the occurrence of ovarian hyperstimulation syndrome (OHSS), which may become severe and even fatal (7, 8). However, Sarkar et al. reported that hyper-physiological serum E2 levels before human chorionic gonadotropin (hCG) triggering had no negative effect on oocyte or embryo quality (9). As a result, local effects of high E2 levels on the maternal environment still remain unclear.

The relationship between serum E2 levels on the day that triggers final oocyte maturation and the outcomes of pregnancy after *in vitro* fertilization (IVF) has been studied for many years, but the results are contradictory (10–13). One recent meta-analysis included three studies and 641 IVF cycles and concluded that there was no difference in clinical pregnancy rates between patients with high and low E2 levels on the hCG trigger day (14).

For ART cycles, the ultimate goal of infertility treatment is successful live births. A study involving 1,141 patients reported an inverted U-shaped smooth curve to explain the association between peak E2 levels and live birth rates (LBRs) in both fresh and frozen embryo transfer (ET) cycles (15). However, no studies with adequate sample size have elaborated on whether the E2 level on the hCG trigger day provides an independent factor affecting LBRs from fresh cycles. Therefore, our objective was to retrospectively evaluate LBRs after fresh ET based on E2 levels on the hCG trigger day of patients, excluding cases exhibiting polycystic ovary syndrome (PCOS).

## Materials and Methods

### Study Population and Design

**Figure 1:**
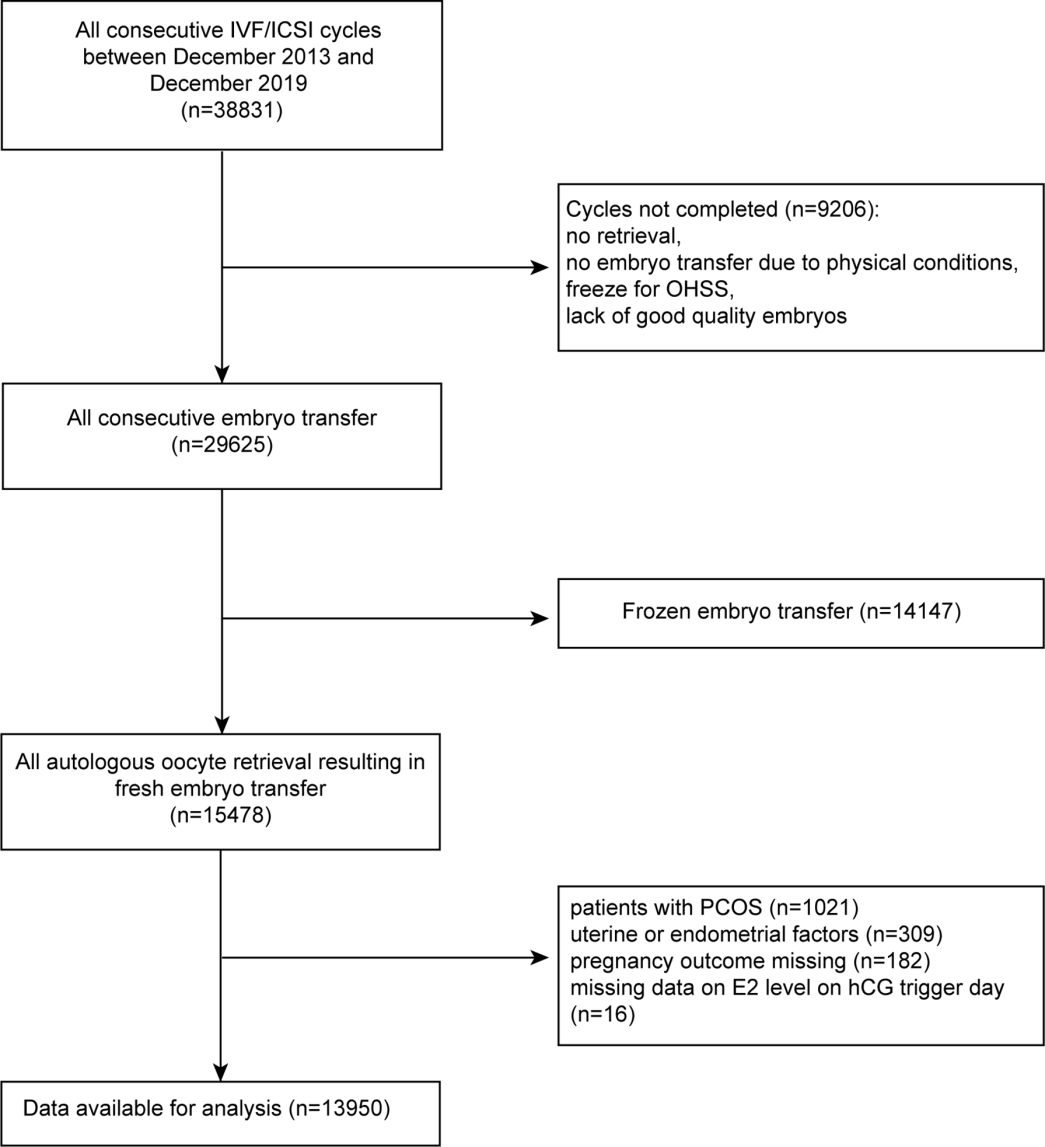
Flow chart for selection of patients from December 2013 to December 2019.

This was a single-center, retrospective study of women who underwent ART at the Reproductive Medicine Center of the Second Hospital of Hebei Medical University between December 2013 and December 2019. A total of 38,831 ET cycles were initially included and 13,950 patients were available after screening. The study details are shown in **Figure 1**. All women were required to have completed ET and had an outcome from the ET by December 2019.

Exclusion criteria included: 1) no embryo transfer at the time of data collection; 2) frozen oocytes; 3) patients diagnosed with PCOS as defined by the Rotterdam diagnostic criteria of PCOS (16); 4) uterine factors affecting pregnancy, including uterine malformations and fibroids, uterus adenomyosis, endometrial polyps, intrauterine adhesions, endometrial tuberculosis history, and uterine effusion; 5) patients lost to follow-up. The remaining 13,950 cases met the acceptance criteria. All patients were observed until the end of pregnancy.

### Descriptive Analysis of Patients’ Characteristics

Baseline demographic indicators included female age (years), male age (years), body mass index (BMI, kg/m^2^) and infertility duration (years). Basal hormone levels included follicle stimulating hormone (FSH, mIU/ml), E2 (pg/ml), progesterone (P, ng/ml) and luteinizing hormone (LH, mIU/ml), measured on the second or third day of menstruation. The parameters for ovarian stimulation included the ovarian stimulation protocol, total gonadotropin dose (IU), gonadotropin duration (days), E2 level (pg/ml) on the hCG trigger day, P level (ng/ml) on the hCG trigger day, antral follicular count (AFC) and number of oocytes retrieved. Embryo parameters included fertilization method, embryo stage, and number of embryos transferred.

### Outcomes

Live birth was defined as the delivery of at least one fetus/baby from the mother (regardless of the length of gestation) and the presence of respiration or any sign of life (e.g., heartbeat, pulsation of the umbilical cord, movement of random muscles, whether the cord had been severed or was still attached to the placenta) after separation from the mother. The main outcome of the study was the LBR per ET and the clinical pregnancy rate (intrauterine pregnancy examined under ultrasound examination showing an intrauterine gestational sac 30 days after transplantation). Early miscarriage was defined as uterine pregnancy loss at < 12 gestational weeks, and late miscarriage was defined as uterine pregnancy loss between 12 and 28 gestational weeks. Pregnancy loss rate = pregnancy losses/all studied cycles.

### Treatment Procedures

For the gonadotropin releasing hormone agonist (GnRH-a) protocol, the patient was given 0.1 mg/day Decapeptyl (Ferring AG, Dübendorf, Switzerland) from the mid-luteal phase. After approximately 14 days, when down-regulation standards were met (LH < 5 IU/L, E2 < 50 pg/L, FSH < 5 IU/L, thickness of endometrium < 5 mm, follicle diameter < 5 mm and no functional ovarian cyst) after serum endocrinology and transvaginal ultrasound examinations, gonadotropin was administered until the hCG trigger day. For the prolonged GnRH agonist protocol, the patient was given 3.75 mg triptorelin (Ipsen Pharma Biotech, Signes, France) on the day 2 of the menstrual cycle. Then 28–31 days later, gonadotrophin was injected when pituitary-ovarian suppression was confirmed and the ovarian response was monitored by vaginal ultrasound and measurement of serum hormone levels during treatment.

The GnRH antagonist (GnRH-ant) protocol started administration from day 2 or 3 of menstruation, followed by daily injections of 0.25 mg Cetrotide (Baxter Oncology GmbH, Frankfurt, Germany) once the leading follicle reached 14 mm diameter and until the hCG trigger day.

The hCG trigger injection was 8000–10000 IU hCG (Lishenbao, Livzon Pharmaceutical Co., Ltd.) or 250 µg recombinant human follitropin alfa (MerckSerono S.p.A, Geneva, Switzerland). The trigger day occurred when one dominant follicle had > 18 mm diameter, or > three follicles had > 17 mm diameter combined with appropriate serum hormone levels. Patients did blood tests with an empty stomach on the hCG trigger day.

At 36–37 h after the hCG trigger was administered, posterior fornix puncture and oocyte retrieval were performed under the guidance of vaginal ultrasound. After 72 h, the embryos were graded according to the number of oocytes, degree of fragmentation, oocyte symmetry, multinucleation, and degree of cell fusion. The embryos were selected for transfer according to the embryo grading criteria (17). If the endometrium was also in good condition (thickness ≥ 8 mm; acceptable morphology), a fresh ET cycle could be performed (with no more than two embryos) if there were no contraindications for transfer. Luteal support was started on the day of oocyte retrieval using oral dydrogesterone tablets (Duphaston Helansuwei Pharmaceutical company), 10 mg twice daily, and 8% progesterone sustained-release vaginal gel (Xenoto, Merck Serono, Germany) followed by the consensus (18). The doses of estrogen and progesterone remained unchanged until the blood β-hCG level was tested 14 days after ET.

### Statistical Methods

Continuous variables were expressed as the mean ± standard deviation if they exhibited a normal distribution and were expressed as the median with quartile if they exhibited a skewed distribution. The number of cases (N) and occurrence percentage (%) were used for categorical variables. Comparison between groups was performed using ANOVA (normally distributed continuous variables), chi-square (categorical variables) or Kruskal-Wallis (non-normally distributed continuous variables) tests to show the difference of baseline characteristics with different pregnancy outcomes, as shown in **Table 1**. We also compared groups with different outcomes in **Table 2** using t-test (normally distributed continuous variables), chi-square (categorical variables) or Mann-Whitney (non-normally distributed continuous variables). Univariate analysis was used to assess whether covariates were segmentally associated with LBRs (**Table 3**).

**Table 1:** Demographic characteristics of the participants.

Demographic characteristics	E2 on hCG trigger day (ng/ml)			*P* value
	Tertile1: 0.02-2.26	Tertile2: 2.27-3.73	Tertile3: 3.74-5.16	
N	4648	4652	4650	
Female age (years)	32.3 ± 5.2	30.5 ± 4.6	29.8 ± 4.2	<0.001
Male age (years)	33.0 ± 5.7	31.3 ± 5.1	30.7 ± 4.7	<0.001
Infertility duration (years)	3.0 (2.0-6.0)	3.0 (2.0-5.0)	3.0 (2.0-5.0)	<0.001
BMI (kg/m^2^)	23.89 ± 3.68	23.32 ± 3.49	22.73 ± 3.50	<0.001
Basal FSH (mIU/ml)	8.61 ± 3.95	7.71 ± 3.24	7.31 ± 2.74	<0.001
Basal E2 (pg/ml)	31.16 (16.00-48.58)	28.74 (13.23-46.00)	33.00 (18.83-48.55)	<0.001
Basal P (ng/ml)	0.60 (0.37-0.89)	0.58 (0.34-0.87)	0.64 (0.39-0.95)	<0.001
Basal LH (mIU/ml)	3.77 (2.75-5.27)	4.06 (2.97-5.51)	4.48 (3.32-6.10)	<0.001
AFC	9.63 ± 4.89	12.17 ± 5.01	13.60 ± 4.91	<0.001
E2 on hCG trigger day (ng/ml)	1.36 ± 0.56	2.99 ± 0.36	4.70 ± 0.39	<0.001
P on hCG trigger day (ng/ml)	0.94 ± 0.71	1.17 ± 0.43	1.33 ± 0.53	<0.001
Endometrium thickness on hCG trigger day (mm)	10.60 ± 2.11	10.91 ± 2.12	10.89 ± 1.98	
Infertility type				<0.001
Primary infertility	2360 (50.7%)	2517 (54.1%)	2630 (56.6%)	
Secondary infertility	2288 (49.3%)	2135 (45.9%)	2020 (43.4%)	
Stimulation protocol				<0.001
GnRH-agonist protocol	1325 (28.5%)	2318 (49.8%)	2763 (59.4%)	
Prolonged GnRH-agonist protocol	1048 (22.5%)	1265 (27.2%)	1166 (25.1%)	
GnRH-antagonist protocol	1351 (29.1%)	555 (11.9%)	469 (10.1%)	
Natural cycle	38 (0.8%)	31 (0.7%)	1 (0.0%)	
Data missing	886 (19.1%)	483 (10.4%)	251 (5.4%)	
Gn total dose (IU)	2657.99 ± 1508.89	2647.65 ± 1423.09	2554.66 ± 862.87	<0.001
Gn duration (day)	10.06 ± 2.72	10.74 ± 2.37	11.01 ± 1.95	<0.001
Method of fertilization				<0.001
IVF	3821 (82.3%)	3730 (80.2%)	3630 (78.1%)	
ICSI	808 (17.4%)	903 (19.4%)	1005 (21.6%)	
IVF&ICSI	15 (0.3%)	16 (0.4%)	13 (0.3%)	
Number of oocytes retrieved	5.56 ± 3.32	9.43 ± 4.33	12.42 ± 4.56	<0.001
Embryo stage				0.031
Cleavage stage	4595 (99.8%)	4595 (99.7%)	4604 (99.6%)	
Blastocyst stage	7 (0.2%)	14 (0.3%)	21 (0.4%)	
number of embryos transferred				<0.001
1	758 (16.3%)	278 (6.0%)	131 (2.8%)	
2	3493 (75.2%)	3937 (84.6%)	4273 (91.9%)	
3	397 (8.5%)	437 (9.4%)	246 (5.3%)	
Biochemical pregnancy	2147 (46.2%)	2513 (54.0%)	2519 (54.2%)	<0.001
Clinical pregnancy	1869 (40.2%)	2198 (47.3%)	2236 (48.1%)	<0.001
Pregnancy loss	434 (20.6%)	448 (18.1%)	418 (16.8%)	0.004
Early miscarriage	283 (65.2%)	278 (62.1%)	262 (62.7%)	
Late miscarriage	151 (34.8%)	170 (38.0%)	156 (37.3%)	
Live birth	1667 (35.9%)	2020 (43.4%)	2062 (44.3%)	<0.001

E2, estradiol; hCG, human chorionic gonadotropin; BMI, body mass index; FSH, follicle stimulating hormone; P, progesterone; LH, luteinizing hormone; AFC, antral follicular count; GnRH, gonadotropin releasing hormone; Gn, gonadotropin; IVF, in vitro fertilization; ICSI, intracytoplasmic single sperm injection.

Normal distribution of data was presented as mean ± standard deviation; nonnormal distribution of data was presented as median (interquartile range) and categorical data using number (percentage).

Numbers that do not add up to 100% are attributed to missing data.

**Table 2:** Demographic characteristics of the participants stratified by outcome.

Demographic characteristics	No live birth	Live birth	*P* value
N	8201	5749	
Female age (years)	31.50 ± 5.11	29.99 ± 4.16	<0.001
Male age (years)	32.24 ± 5.62	30.82 ± 4.66	<0.001
Infertility duration (years)	3.0 (2.0-6.0)	3.0 (2.0-5.0)	<0.001
BMI (kg/m2)	23.35 ± 3.65	23.25 ± 3.48	0.138
Basal FSH (mIU/ml)	7.98 ± 3.26	7.72 ± 3.55	<0.001
Basal E2 (pg/ml)	31.00 (15.81-48.00)	31.00 (15.85-47.56)	0.833
Basal P (ng/ml)	0.61 (0.37-0.90)	0.61 (0.37-0.91)	0.191
Basal LH (mIU/ml)	4.09 (2.97-5.59)	4.15 (3.02-5.66)	0.537
AFC	11.41 ± 5.25	12.47 ± 5.06	<0.001
E2 on hCG trigger day (ng/ml)	29.19 ± 14.74	31.50 ± 13.68	<0.001
P on hCG trigger day (ng/ml)	1.15 ± 0.63	1.13 ± 0.53	0.07
Endometrium thickness on hCG trigger day (mm)	10.66 ± 2.08	10.99 ± 2.05	<0.001
Infertility type			0.027
Primary infertility	4349 (53.0%)	3158 (54.9%)	
Secondary infertility	3852 (47.0%)	2591 (45.1%)	
Stimulation protocol			<0.001
GnRH-agonist protocol	3537 (43.1%)	2869 (49.9%)	
Prolonged GnRH-agonist protocol	1850 (22.6%)	1629 (28.3%)	
GnRH-antagonist protocol	1620 (19.7%)	755 (13.1%)	
Natural cycle	55 (0.7%)	15 (0.3%)	
Data missing	1139 (13.9%)	481 (8.4%)	
Gn total dose (IU)	2657.29 ± 1505.13	2567.04 ± 922.06	<0.001
Gn duration (day)	10.53 ± 2.48	10.72 ± 2.28	<0.001
Method of fertilization			<0.001
IVF	6654 (81.2%)	4527 (78.8%)	
ICSI	1523 (18.2%)	1193 (20.8%)	
IVF&ICSI	19 (0.2%)	25 (0.4%)	
Number of oocytes retrieved	8.64 ± 5.02	9.85 ± 4.81	<0.001
Embryo stage			0.916
Cleavage stage	8100 (99.7%)	5694 (99.7%)	
Blastocyst stage	25 (0.3%)	17 (0.3%)	
number of embryos transferred			<0.001
1	970 (11.8%)	197 (3.4%)	
2	6547 (79.8%)	5156 (89.7%)	
3	684 (8.4%)	396 (6.9%)	

BMI, body mass index; FSH, follicle stimulating hormone; E2, estradiol; P, progesterone; LH, luteinizing hormone; AFC, antral follicular count; hCG, human chorionic gonadotropin; GnRH, gonadotropin releasing hormone; Gn, gonadotropin; IVF, in vitro fertilization; ICSI, intracytoplasmic single sperm injection.

Normal distribution of data was presented as mean ± standard deviation; nonnormal distribution of data was presented as median (interquartile range) and categorical data using number (percentage).

**Table 3:** Crude association of live birth with baseline characteristics and intervention and embryo parameters.

	Statistics	RR (95%Cl)	*P* value
Female age (years)			
≤35	11588 (83.1%)	Ref	
>35	2361 (16.9%)	0.573 (0.531, 0.614)	<0.001
Male age (years)			
≤35	11056 (79.4%)	Ref	
>35	2869 (20.6%)	0.672 (0.635, 0.716)	<0.001
Infertility duration (year)			
≤1	2664 (19.4%)	Ref	
>1	11081 (80.6%)	0.932 (0.899, 0.976)	<0.001
BMI (kg/m^2^)			
≤24	8420 (62.8%)	Ref	
>24	4986 (37.2%)	0.966 (0.929, 1.009)	0.063
Basal FSH (mIU/ml)			
≤15	12530 (97.8%)	Ref	
>15	289 (2.2%)	0.615 (0.499, 0.731)	<0.001
Basal E2 (pg/ml)			
≤50	9975 (77.8%)	Ref	
>50	2848 (22.2%)	0.973 (0.925, 1.021)	0.161
Basal P (ng/ml)			
≤0.9	8295 (75.2%)	Ref	
>0.9	2743 (24.8%)	1.013 (0.963, 1.062)	0.306
Basal LH (mIU/ml)			
≤10	12148 (95.1%)	Ref	
>10	625 (4.9%)	1.000 (0.907, 1.101)	0.495
AFC			
≤8	3037 (27.1%)	Ref	
>8, ≤12	3853 (34.4%)	1.109 (1.029, 1.190)	<0.01
>12	4316 (38.5%)	1.166 (1.080, 1.261)	<0.01
Endometrium thickness on hCG trigger day (mm)			
≤7	531 (3.8%)	Ref	
>7	13299 (96.2%)	1.437 (1.263, 1.661)	<0.001
E2 on hCG trigger day (ng/ml)			
≤2.26	4648 (33.3%)	Ref	
>2.26, ≤3.73	4652 (33.4%)	1.211 (1.150, 1.273)	<0.001
>3.73	4650 (33.3%)	1.236 (1.178, 1.301)	<0.001
P on hCG trigger day (ng/ml)			
≤0.9	4622 (33.1%)	Ref	
>0.9, ≤3	9295 (66.6%)	0.983 (0.945, 1.023)	0.217
>3	33 (0.3%)	0.800 (0.434, 1.190)	0.150
Infertility type			
Primary infertility	7507 (53.8%)	Ref	
Secondary infertility	6443 (46.2%)	0.956 (0.920, 0.996)	0.150
Stimulation protocol			
GnRH-agonist protocol	6406 (45.9%)	Ref	
Prolonged GnRH-agonist protocol	3479 (24.9%)	1.046 (0.997, 1.091)	<0.05
GnRH-antagonist protocol	2375 (17.0%)	0.710 (0.663, 0.756)	<0.001
Natural cycle	70 (0.5%)	0.479 (0.266, 0.710)	<0.001
Data missing	1620 (11.6%)	0.663 (0.611, 0.714)	<0.001
Gn total dose (IU)			
≤2174	4494 (32.2%)	Ref	
>2174, ≤2840	4692 (33.6%)	0.971 (0.924, 1.018)	0.106
>2840	4763 (34.2%)	0.879 (0.837, 0.925)	<0.001
Gn duration (day)			
T1 (≤10)	4181 (30.0%)	Ref	
T2 (>10, ≤11)	2913 (20.9%)	1.091 (1.032, 1.155)	<0.001
T3 (>11)	6855 (49.1%)	1.107 (1.059, 1.158)	<0.001
Method of fertilization			
IVF	11218 (80.2%)	Ref	
ICSI	2726 (19.5%)	1.085 (1.034, 1.141)	<0.001
IVF&ICSI	44 (0.3%)	1.403 (0.039, 1.775)	<0.05

BMI, body mass index; FSH, follicle stimulating hormone; LH, luteinizing hormone; AFC, antral follicular count; Gn, gonadotropin; P, progesterone; hCG, human chorionic gonadotropin; GnRH, gonadotropin releasing hormone; Gn, gonadotropin; IVF, in vitro fertilization; ICSI, intracytoplasmic single sperm injection.

Covariates were included as potential confounders in the final models if they changed the estimates of E2 levels by more than 10% with LBRs or were significantly associated with LBRs. Clinical experience and studies published in recent years were taken into consideration. The following covariates were selected *a priori* on the basis of established associations and/or plausible biological relations and tested: female age, male age, infertility duration, BMI, basal FSH, basal LH, AFC, P on the hCG trigger day, endometrium thickness on the hCG trigger day, stimulation protocol, total dose of gonadotropin and gonadotropin duration, method of fertilization and type of infertility (primary infertility or secondary infertility). Screening processes were shown in **Supplementary Tables 1** and **2**. We classified these variables according to their clinical significance (19–21).

Multivariable logistic regression was used for multivariable analysis. Three models were constructed, as shown in **Table 4**: crude regression estimates without adjusted covariates; model I, estimates adjusted for female age and BMI; model II, estimates adjusted for all covariates. We adjusted the covariates that were nonlinearly associated with the outcome by smooth fitting. In model I, the degree of freedom of the curve fitting adjusted for female age was 7.677. Moreover, the degree of freedom of the smooth fitting in model II adjusted for female age was 7.613 and for AFC was 2.886.

**Table 4:** Multivariable regression analysis models examining the association between E2 level on hCG trigger day and LBR.

LBR	Crude RR (95%CI)	*P* value	Model I RR (95%CI)	*P* value	Model II RR (95%CI)	*P* value
E2 on hCG trigger day (1000 pg/ml)	1.067 (1.054, 1.082)	<0.001	1.041 (1.026,1.056)	<0.001	1.027 (1.007, 1.049)	<0.05
Patients were equally divided into three groups according to E2 levels				<0.001		
Low	Ref		Ref		Ref	
Intermediate	1.211 (1.150, 1.273)	<0.001	1.126 (1.068, 1.185)	<0.001	1.077 (1.008, 1.151)	<0.05
High	1.236 (1.178, 1.301)	<0.001	1.133 (1.077, 1.192)	<0.001	1.093 (1.019, 1.179)	<0.05
Three groups treated as continuous variables	1.105 (1.035, 1.195)	<0.001	1.061 (1.009, 1.113)	<0.001	1.042 (1.002, 1.166)	<0.05

Model I: adjusted for female age (Smooth) and BMI. The general additive model was applied as the adjusted Model I.

Model II: following factors were adjusted: female age (Smooth), male age, infertility duration, BMI, basal FSH, basal LH, AFC (Smooth), infertility type, stimulation protocol, Gn total dose, Gn duration, Endometrium thickness on hCG trigger day, P on hCG trigger day, method of fertilization. The general additive model was applied as the adjusted Model II.

Sensitivity analysis examined the stability of the results: (1) Stratification analysis was performed and each stratification was adjusted for all covariates except the stratification factor itself. Interaction analysis was performed for female age (≤35 and >35 years), BMI (≤24 and >24 kg/m^2^), infertility duration (≤1 and >1 year), AFC (≤8, >8, ≤12 and >12), endometrium thickness on the hCG trigger day (≤7 and >7 mm), infertility type (primary fertility and secondary fertility), stimulation protocol (GnRH-agonist protocol, prolonged GnRH-agonist protocol, GnRH-antagonist protocol, and natural cycle), and method of fertilization (IVF, ICSI and IVF&ICSI) (**Table 6**). (2) Serum E2 levels were converted into a categorical variable according to the tertile to examine the results of E2 levels and determine the possibility of curvilinear association (**Table 4**).

**Figure 2:**
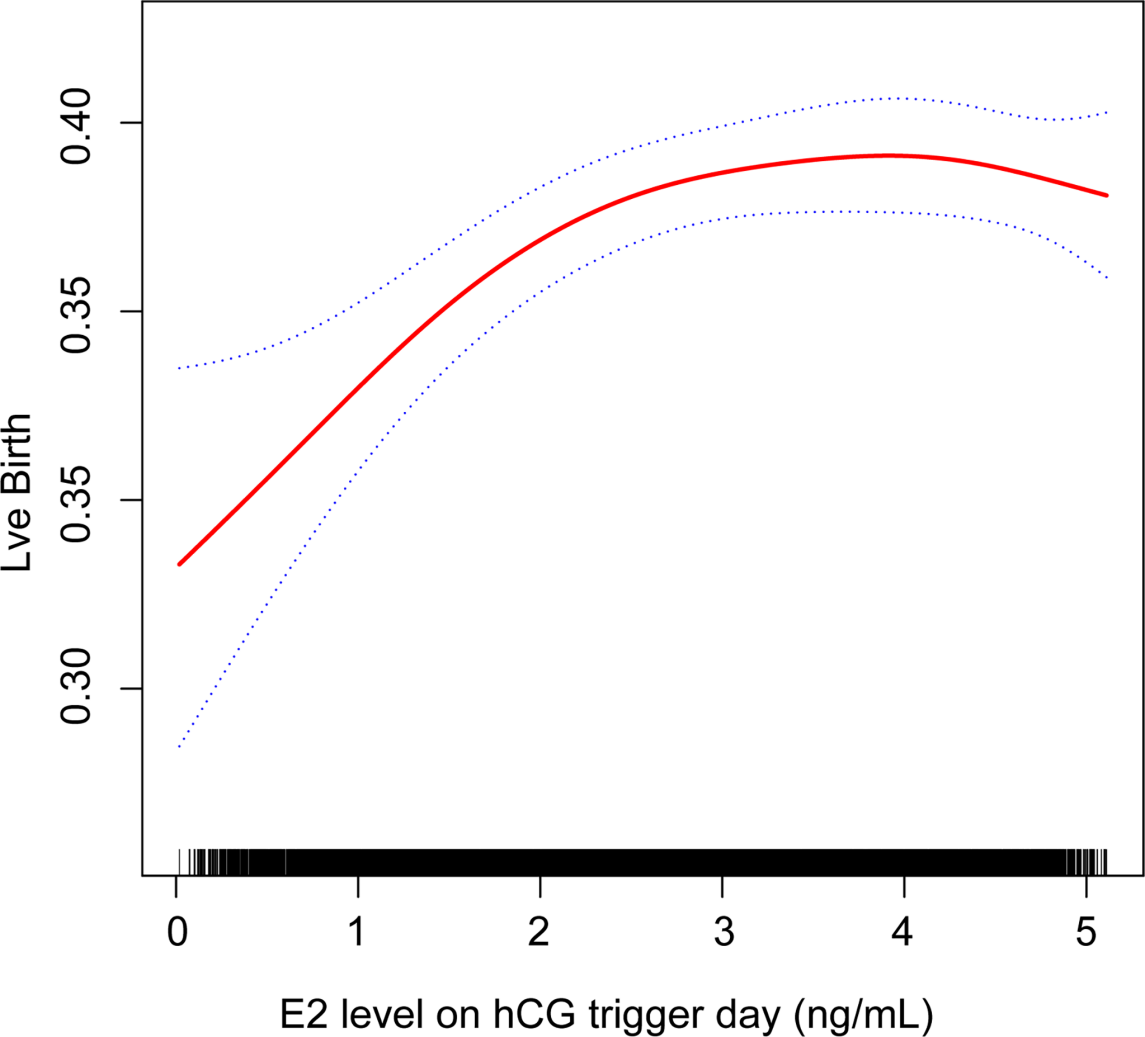
Association between live birth and estradiol (E2) level on the hCG trigger day. A threshold, nonlinear association between live birth and E2 level was found in a generalized additive model (GAM). Solid red line represents the smooth curve fit between these two variables. Blue lines represent the 95% of confidence interval from the fit.

Generalized additive models were used to investigate the non-linear relationship between E2 levels and LBRs. The degree of freedom was determined by the minimum GCV method, which in our study is 2.4885. A two-piecewise linear regression model was used to examine the threshold effect of the E2 level on LBR according to the smoothed plot (**Figure 2**). The threshold level of E2 at which the relationship between LBRs and E2 levels began to change was determined using a recurrence method. An inflection point was moved along a predefined interval to detect the inflection point that gave the maximum likelihood of the model (**Table 5**). To ensure the robustness of data analysis, the relative risk (RR) value represents the change in the ratio of LBRs for every 1 ng/ml increase in E2 level in this study. *P* < 0.05 was used for statistical significance. All statistical analyses were performed using statistical software packages R (http://www.R-project.org, The R Foundation) and EmpowerStats software (http://www.mpowerstats.com, X&Y Solution, Inc., Boston, MA).

**Table 5:** The result of two-piecewise linear regression model.

Models	Adjusted RR (95%Cl)	*P* value
Model I		
One line slope	1.027 (1.007, 1.049)	<0.05
Model II		
Inflection point	2.1	
< 2.1	1.188 (1.057, 1.334)	<0.01
> 2.1	1.002 (0.971, 1.032)	0.429
LRT test	0.006	

Model I, linear analysis; Model II, nonlinear analysis. LRT test, logarithmic likelihood ratio test (p value < 0.05 indicates that Model II is significantly different from Model I, which indicates a nonlinear relationship); adjustment variables: female age (Smooth), male age, infertility duration, BMI, basal FSH, basal LH, AFC (Smooth), infertility type, stimulation protocol, Gn total dose, Gn duration, Endometrium thickness on hCG trigger day, P on hCG trigger day, method of fertilization.

BMI, body mass index; FSH, follicle stimulating hormone; LH, luteinizing hormone; AFC, antral follicular count; Gn, gonadotropin; P, progesterone; hCG, human chorionic gonadotropin.

## Results

### Descriptive Analysis of Study Population

Of a total of 38,831 fresh/frozen ET cycles from December 2013 to December 2019, 13,950 cycles were finally included (**Figure 1**). **Table 1** shows the overall demographic data of the study cohort, including baseline clinical and biochemical characteristics, ovarian stimulation parameters, embryo parameters, and pregnancy outcomes. The mean age of the 13,950 cycles in this study was 30.9 ± 4.8 years for women and 31.7 ± 5.3 years for men. There were 7,507 cases (53.81%) of primary infertility and 6,443 cases (46.19%) of secondary infertility. The mean E2 and P levels on the hCG trigger day were 3.01 ± 1.44 ng/ml and 1.15 ± 0.59 ng/ml, respectively. There were 5,749 (41.21%) live births, and the proportion was a little higher than that reported in another study (22). Compared with the two groups with lower E2 levels on the hCG trigger day, participants in the elevated E2 group were younger, had a lower BMI, higher basal LH and lower basal FSH, had an increased AFC on the hCG trigger day, required lower doses of gonadotropins for stimulation, had more retrieved oocytes, and had a higher LBR. **Table 2** demonstrates the parameters for the different pregnancy outcomes (live birth or not). The differences were statistically significant except for BMI, basal E2, basal P, basal LH, P on the hCG trigger day, and embryo stage.

### Factors in the Participants Associated With Live Birth

**Table 3** presents the association between live birth and baseline characteristics, parameters for ovarian stimulation, and embryo parameters chosen as covariates. Consistent with the available literature, higher endometrium thickness on the hCG trigger day, increased AFCs, extended gonadotropin duration, a prolonged GnRH-agonist protocol, and intracytoplasmic sperm injection (ICSI) and IVF methods of fertilization were each associated with increased LBRs, whereas higher female age, longer infertility duration, higher basal FSH, increased total dose of gonadotropin, GnRH-antagonist protocol, natural cycle, and data missing on stimulation protocols were associated with decreased LBRs. Although few patients underwent natural cycles in this population, this cycle was significantly associated with reduced LBR compared with the GnRH-agonist protocol [RR 0.479, 95% confidence interval (CI) 0.266, 0.710]. The BMI, basal E2, basal P, basal LH, infertility type, and level of P on the hCG trigger day were not significantly associated with LBRs (**Table 3**).

### Multivariable and Stratification Analysis

As shown in **Table 4**, a positive association was found between E2 levels on the hCG trigger day and LBRs in the crude model with every 1 ng/ml increase of E2 (RR 1.067, 95% CI 1.054, 1.082). Compared with crude regression analyses, the associations in the multivariable regression analyses did not change markedly after adjusting for female age and BMI in model I (RR 1.041, 95% CI 1.026, 1.056), and adjusting for all covariates in model II (RR 1.027, 95% CI 1.007, 1.049). The positive relationship remained. The non-equally spaced change in the categorical variable of E2 levels indicated the possibility of a nonlinear relationship between E2 levels and LBRs.

The interaction analysis was also included in the model showing that the majority of stratification factors failed to have an interaction effect with E2 levels on LBRs (**Table 6**). Interestingly, the results implicated that the effect of E2 levels on LBRs was different between subgroups with different AFCs. In the subgroup with a low AFC, the LBR was positively associated with E2 levels on the hCG trigger day; specifically, the LBR increased by nearly 5.2% with every 1 ng/ml increase of E2 (RR 1.052, 95% CI 1.004, 1.102). Although the effect value was much smaller, the P value showed significance.

**Table 6:** Stratification analysis in different subgroups.

Stratification characteristics	N	LBR		*P* for interaction
		Adjusted RR (95%Cl)	*P* value	
Female age				0.290
≤35	11588	1.027 (1.006, 1.051)	<0.01	
>35	2361	1.012 (0.950, 1.093)	0.346	
Infertility duration (year)				0.385
≤1	2664	1.039 (0.994, 1.096)	0.052	
>1	11081	1.026 (1.003, 1.048)	<0.05	
BMI (kg/m^2^)				0.647
≤24	8420	1.022 (0.996, 1.051)	<0.05	
>24	4986	1.035 (1.000, 1.076)	<0.05	
AFC				< 0.01
≤8	3037	1.052 (1.004, 1.102)	0.015	
>8, ≤12	3853	1.011 (0.974, 1.052)	0.297	
>12	4316	1.014 (0.984, 1.045)	0.174	
Endometrium thickness on hCG trigger day (mm)				0.108
≤7	531	1.150 (0.997, 1.383)	<0.05	
>7	13299	1.026 (1.006, 1.048)	<0.01	
Infertility type				0.542
Primary infertility	7507	1.005 (1.000, 1.010)	0.057	
Secondary infertility	6443	1.029 (1.000, 1.061)	0.169	
Stimulation protocol				0.963
GnRH-agonist protocol	6406	1.022 (0.993, 1.053)	0.073	
Prolonged GnRH-agonist protocol	3479	1.022 (0.987, 1.055)	0.115	
GnRH-antagonist protocol	2375	1.015 (0.951, 1.087)	0.340	
Natural cycle	70	——	——	
Data missing	1620	1.122 (1.019, 1.234)	0.014	
Method of fertilization				0.627
IVF	11181	1.030 (1.008, 1.055)	<0.05	
ICSI	2716	1.015 (0.996, 1.066)	0.236	
IVF&ICSI	44	——	——	

BMI, body mass index; AFC, antral follicular count; Gn, gonadotropin; P, progesterone; GnRH, gonadotropin releasing hormone; IVF, in vitro fertilization; ICSI, intracytoplasmic single sperm injection.

The following factors, except the stratification factor itself, were adjusted in the multivariable analysis: female age (Smooth), male age, BMI, infertility duration, basal FSH, basal LH, AFC (Smooth), infertility type, stimulation protocol, Gn total dose, Gn duration, Endometrium thickness on hCG trigger day, P on hCG trigger day, method of fertilization.

### Nonlinear Relationship Between Serum E2 Levels and LBR

Curvilinear relationships were examined for continuous variables by utilizing the two-piecewise linear regression model. This research found a curvilinear relationship between E2 levels and LBRs after adjusting for covariates, including baseline demographic indicators, parameters for ovarian stimulation, and embryo parameters (**Figure 2**). The inflection point was calculated as 2.1 ng/ml E2 by a recursive algorithm (**Table 5**). The analysis of threshold effects indicated that LBRs increased with every 1 ng/ml E2 when the concentration of E2 was lower than 2.1 ng/ml (RR 1.188, 95% CI 1.057, 1.334); however, there was no significant association between E2 levels and LBRs (RR 1.002, 95% CI 0.971, 1.032) when the concentration of E2 was higher than 2.1 ng/ml.

## Discussion

With a two-piecewise linear regression model, our results showed that when serum E2 levels were lower than 2.1 ng/ml on the hCG trigger day, the LBR increased by about 18.8% with every 1 ng/ml increase in E2. When serum E2 levels were beyond 2.1 ng/ml on the hCG trigger day, there was no significant association between E2 levels and LBRs.

Follicular fluid is composed of factors secreted by granulosa and follicular cells, and plasma permeate. This fluid provides the microenvironment for the development and differentiation of the oocyte-cumulus-corona complex., granulosa cells, and follicular cells (23). The development of oocytes depends on changes in various hormones and growth factors in follicular fluid, as well as the interaction between these substances and oocytes and granulosa cells (24, 25). Estradiol is mainly secreted periodically by follicular granulosa cells (26). The level of serum E2 can indirectly reflect the level of E2 in follicular fluid (27), and the level of E2 in follicular fluid is positively related to the maturity of the oocyte. Therefore, serum E2 levels are closely related to oocyte production and maturity. However, the relationship between E2 and live birth has not been clearly demonstrated.

This study found a positive relationship between LBRs and serum E2 levels on the hCG trigger day when E2 concentrations were lower than 2.1 ng/ml, which was consistent with previous findings: Foroozanfard et al. conducted a study of 128 IVF cycles and showed that pregnancy rates and E2 levels were positively correlated (28); with a sample size similar to the above study, Wei et al. demonstrated that patients undergoing IVF/ICSI cycles with lower serum E2 levels on the hCG trigger day had a low clinical pregnancy rate (29); Siddhartha et al. reported a positive association of LBRs with E2 levels (in the range of 300–4000 pg/ml) using logistic regression analysis of 89 ICSI cycles (30); Li et al. reported a steady growing trend for increased LBR when E2 levels were lower than 5000 pg/ml, including in fresh IVF cycles of 1771 infertile patients (31); and a recent study found that when E2 levels were less than 2185 pg/ml, the cumulative LBR increased by about 12% for every 100 pg/ml increase in E2 level in patients, excluding those exhibiting PCOS (15). Compared with the above studies, the current study had a larger sample size and focused on patients without uterine factors and not experiencing PCOS. In addition, some researchers used E2/follicle number as an observation indicator and found that low E2/follicle number ratios were associated with low oocyte retrieval rates and a decreased possibility of live birth because of single and triple pronucleus formation (32).

The platform between E2 levels and LBRs indicated that when serum E2 levels reached a certain concentration on the day of the hCG, there was no longer a significant beneficial relationship. This association was also supported by the result of stratification analysis based on AFCs. The LBR was significantly increased with elevated E2 levels on the hCG trigger day for patients having the lowest AFCs, and this impact of E2 on LBR was intrinsically different from people with higher AFCs on the hCG trigger day (*P* for interaction < 0.01). The finding that AFC was closely associated with serum E2 levels on the hCG trigger day was consistent with our main result. The positive impact of E2 disappeared when a patient’s ovaries over-responded. Several studies reported similar findings: Ze et al. found that high E2 levels (> 19124 pmol/L) did not influence pregnancy rates or LBRs in 2921 patients (10); two similar studies examined 106 and 274 patients, respectively, and concluded that high E2 levels were not harmful to pregnancy outcomes (11, 12); another study of 207 patients suggested that the E2 concentration on the hCG trigger day has nothing to do with pregnancy rates, but had potentially deleterious effects on endometrial receptivity because despite improved embryo quality, there was no accompanying increase in pregnancy rate; Zhang et al. found that a downward trend for LBRs was consistent with the results of freeze-thaw ET cycles when E2 levels declined, implying that a negative relationship between LBRs and E2 levels was mostly from frozen ET cycles and not fresh cycles (15). Many studies have demonstrated a negative correlation between high E2 levels on the hCG trigger day and pregnancy outcomes. Jianhua et al. found that the E2 level to oocyte ratio was associated with lower pregnancy and implantation rates (33). It also pointed out that these poor reproductive results may be due to the high E2 concentration, which adversely affected the receptivity of the endometrium. This explanation was also suggested in other studies, showing that much higher E2 levels were not favorable for implantation and pregnancy rates (5, 6). Moreover, a more recent study demonstrated that no significant effect on LBR was found in normal responders with letrozole co-treatment reducing E2 level (34). This may be due to the fact that letrozole not only inhibits the production of estrogen, but also affects the recruitment of follicles neutralizing the negative impact of reduced E2 both through enhanced serum levels of gonadotrophins and because of the endogenous acidic isoform of FSH, which is lacking in the recombinant exogenous FSH (35).

According to previous reports, when E2 levels reach a certain threshold, the adverse effects of high E2 levels on endometrial receptivity may be related to weakened endometrial blood flow (36, 37), enhanced uterine contractility (38), interference with glycogen synthesis and secretion (39), intrauterine histopathological changes of nuclear amygdala channels (40, 41), and changes in mitochondrial function (26, 42). High E2 concentrations also promoted the apoptosis of endometrial gland cells in a positive feedback pathway, further damaging the receptivity of the endometrium (43). Elevated E2 concentrations can also affect endometrial receptivity by altering the expression of C3, plasminogen, and kininogen-1 (44). However, the exact mechanism by which high E2 levels are associated with reduced LBRs is unclear. Basic experiments are required to further explore the underlying mechanisms involved in the relationship between high E2 levels and LBRs.

It is worth mentioning that pregnancy outcomes may be affected by the number of transferred embryos. Theoretically, compared with the transfer of two or three embryos, for cases with the transfer of a single embryo the potential benefit of higher E2 levels may not be conspicuous. This is because women with low serum E2 levels can still produce high-quality embryos on the day when final oocyte maturation is triggered, so the ratio between the number of oocytes and embryos transferred is small. This study focused on live births per ET cycle rather than the cumulative live birth rate per oocytes retrieval. Therefore, the potential benefits of high E2 levels cannot be measured. However, the potential benefits of high E2 levels may be balanced by the negative correlation between E2 and endometrial receptivity, which can explain our results.

This study had several strengths. First, this work revealed a curvilinear relationship between estradiol levels on the day of the hCG administration and LBR, and this relationship may contribute to deciding whether to perform ET under such a hormone environment. Generalized additive models specialize in coping with curvilinear relationships and allow nonparametric smoothing and configuration of a regression spline. Second, this study used strict statistical analysis to minimize interference by controlling potential confounding factors. Third, subgroup analysis showed that the current results remained consistent under the influence of different factors. Additionally, the sample size was larger than the majority of similar studies.

This study also had several limitations. This was a retrospective study and there were a few differences in baseline characteristics. First, recall bias is innate and there is the possibility for confusion regarding data from patients’ medication history. Second, although a number of population baseline variables and clinical covariates were included in the adjustment, this study cannot exclude the possibility of residual confounding variables, such as causes of infertility (e.g., tubal factors, mental health factors, and immune factors) and anti-Müllerian hormone levels. Therefore, additional well-designed and randomized control trial studies are needed to further elucidate the association of serum E2 and LBR. In addition, this study was single-center-based and may not be applicable to other centers; more studies are needed to enhance its generalizability.

## Conclusions

Based on our research, the E2 level on the hCG trigger day appears to have a segmental relationship with the LBR. Further prospective studies are required to explore the possible mechanisms and to consolidate the association between serum E2 levels on the hCG trigger day and LBR.

## Data Availability Statement

The original contributions presented in the study are included in the article/**Supplementary Material**. Further inquiries can be directed to the corresponding author.

## Ethics Statement

The studies involving human participants were reviewed and approved by the ethics committee of the Second Hospital of Hebei Medical University. The patients/participants provided their written informed consent to participate in this study.

## Author Contributions

XX, AY contributed to the study design. XX, YH, NC, WW and GH performed data collection, data interpretation and data analysis. NC, WW and GH advised on the conduct and coordination of the study. XX wrote the first draft of the manuscripts. NC obtained funding. All authors contributed to the interpretation of the results and critical revision of the manuscript for important intellectual content and approved the final paper.

## Funding

This study was supported by Natural Science Foundation of Hebei Province (H2019206674) and Natural Science Foundation of Hebei Province (Beijing-Tianjin-Hebei Cooperation Special Project) (H2019206707).

## Conflict of Interest

The authors declare that the research was conducted in the absence of any commercial or financial relationships that could be construed as a potential conflict of interest.

## Publisher’s Note

All claims expressed in this article are solely those of the authors and do not necessarily represent those of their affiliated organizations, or those of the publisher, the editors and the reviewers. Any product that may be evaluated in this article, or claim that may be made by its manufacturer, is not guaranteed or endorsed by the publisher.
