# Supplementary material for: The Association Between Serum Estradiol Levels on hCG Trigger Day and Live Birth Rates in Non-PCOS Patients: A Retrospective Cohort Study
Source: Front Endocrinol (Lausanne). 2022 May 3;13:839773. doi: 10.3389/fendo.2022.839773 (PMC9112425; doi:10.3389/fendo.2022.839773)
Supplement: Supplementary file 1 [file Table_1.docx]

Supplementary Table 1. The relationship between covariates and Y (LB).

| Covariates | N | beta | Se. | exp(beta) | 95%CI Low | 95%CI Upp | P.value |
| --- | --- | --- | --- | --- | --- | --- | --- |
| FSH | 12653 | -0.0128 | 0.0059 | 0.9873 | 0.9758 | 0.9988 | 0.0306 |
| E2 | 12657 | 0.0000 | 0.0003 | 1.0000 | 0.9994 | 1.0007 | 0.9091 |
| P | 10889 | -0.0057 | 0.0046 | 0.9943 | 0.9853 | 1.0033 | 0.2153 |
| LH | 12611 | -0.0024 | 0.0049 | 0.9976 | 0.9881 | 1.0071 | 0.6152 |
| Infertility type | 13407 | 0.1265 | 0.0374 | 1.1348 | 1.0546 | 1.2211 | 0.0007 |
| Stimulation protocol | 13407 | 0.0776 | 0.0435 | 1.0806 | 0.9924 | 1.1767 | 0.0744 |
|  |  | -0.4045 | 0.0537 | 0.6673 | 0.6007 | 0.7414 | <0.0001 |
|  |  | -0.7677 | 0.3071 | 0.4641 | 0.2542 | 0.8472 | 0.0124 |
|  |  | -0.5150 | 0.0624 | 0.5975 | 0.5288 | 0.6752 | <0.0001 |
| Method of fertilization | 13398 | 0.0497 | 0.0447 | 1.0509 | 0.9627 | 1.1472 | 0.2667 |
|  |  | 0.6773 | 0.3123 | 1.9686 | 1.0673 | 3.6307 | 0.0301 |
| Gn total dose | 13406 | 0.0000 | 0.0000 | 1.0000 | 0.9999 | 1.0000 | 0.0659 |
| AFC | 10890 | 0.0249 | 0.0040 | 1.0252 | 1.0172 | 1.0334 | <0.0001 |
| Gn duration | 13406 | 0.0219 | 0.0076 | 1.0221 | 1.0070 | 1.0374 | 0.0040 |
| Endometrium thickness on hCG trigger day | 13295 | 0.0646 | 0.0087 | 1.0667 | 1.0487 | 1.0849 | <0.0001 |
| Male age | 13386 | -0.0080 | 0.0060 | 0.9921 | 0.9804 | 1.0039 | 0.1859 |
| P on hCG trigger day | 13407 | -0.1149 | 0.0351 | 0.8915 | 0.8323 | 0.9549 | 0.0011 |
| Infertility duration | 13207 | -0.0233 | 0.0063 | 0.9770 | 0.9650 | 0.9892 | 0.0002 |

Supplementary Table 2. Introducing covariates in the basic model versus removing them from the full model and observing the change in the regression coefficient of X.
X= E2 level on hCG trigger day (ng/mL)

|  | Basic model | Full model |  |
| --- | --- | --- | --- |
| Covariates | HCGE2100 | HCGE2100 | Selected |
|  | 0.0611 | 0.0066 |  |
| FSH | 0.0549 * | 0.0058 * | Yes |
| E2 | 0.0611 | 0.0073 |  |
| P | 0.0607 | 0.0073 |  |
| LH | 0.0635 | 0.0042 * | Yes |
| Infertility type | 0.0608 | 0.0054 * | Yes |
| Stimulation protocol | 0.0612 | 0.0088 * | Yes |
| Method of fertilization | 0.0611 | 0.0090 * | Yes |
| Gn total dose | 0.0372 * | 0.0104 * | Yes |
| AFC | 0.0549 * | 0.0083 * | Yes |
| Gn duration | 0.0566 | 0.0053 * | Yes |
| Endometrium thickness on hCG trigger day | 0.0609 | 0.0066 | Yes |
| Male age | 0.0911 * | -0.0189 * |  |
| P on hCG trigger day | -0.0030 * | 0.0424 * | Yes |
| Infertility duration | 0.0269 * | 0.0097 * | Yes |

* indicates a change of more than 10% from the starting regression coefficient
